# Stakeholder perspectives on the barriers and facilitators of engagement in healthy lifestyle behaviours in underrepresented adolescents: a focus group study from the European SEEDS project

**DOI:** 10.1186/s12889-024-19419-4

**Published:** 2024-07-25

**Authors:** Annemieke Wargers, Christopher M. Elphick, Famke J. M. Mölenberg, Amandine Senequier, Yannis Manios, Christina Mavrogianni, Claire Murray, Judit Queral, Lucia Tarro, Craig A. Williams, Dimitris Vlachopoulos, Wilma Jansen

**Affiliations:** 1 Department of Public Health, Erasmus MC, University Medical Center Rotterdam, Rotterdam, the Netherlands; 2 Children’s Health and Exercise Research Centre, Department of Public Health and Sport Science, University of Exeter, Exeter, United Kingdom; 3 Department of Nutrition and Dietetics, School of Health Science and Education, Harokopio University, Athens, 17671 Greece; 4 Institute of Agri-food and Life Sciences, University Research & Innovation Center, H.M.U.R.I.C, Hellenic Mediterranean University, Crete, GR-71003 Greece; 5 European Citizen Science Association, c/o Museum für Naturkunde Invalidenstraße 43, 10115 Berlin, Germany; 6 Institut d’Investigació Sanitària Pere Virgili (IISPV), Reus, 43204 Spain; 7 Functional Nutrition, Oxidation, and Cardiovascular Diseases Group (NFOC-Salut), Healthy Environment Chair, Facultat de Medicina i Ciències de la Salut, Universitat Rovira i Virgili, Reus, 43201 Spain; 8 Department of Social Development, City of Rotterdam, Rotterdam, the Netherlands

**Keywords:** Adolescents, Socioeconomic status, Thematic analysis, Physical activity, Diet, Theory of Planned Behaviour, Stakeholders

## Abstract

**Background:**

Obesity in adolescence has increased in the last decades. Adolescents fail to meet the recommended guidelines for physical activity (PA) and healthy diet. Adolescents with a low socioeconomic status (SES) particularly seem to have fewer healthy lifestyle behaviours. The European Science Engagement to Empower aDolescentS (SEEDS) project used an extreme citizen science approach to develop and implement healthy lifestyle behaviour interventions in high schools. As part of this project, key stakeholders were invited to reflect on the intentions of adolescents to engage in healthy lifestyle behaviours. The aim of this study was to gain stakeholder insights into the barriers and facilitators to healthy lifestyle behaviours of adolescents from low SES areas and on the possible role of these stakeholders in facilitating healthy lifestyle behaviours.

**Methods:**

Six semi-structured focus groups were conducted in four European countries with 28 stakeholders from different settings (schools, community, and government), like teachers, policy advisors and youth workers. The theoretical framework of focus groups was based on the Theory of Planned Behaviour. The main questions of the focus groups were centred on PA and healthy diet. The focus groups were qualitatively analysed in NVivo using thematic analysis to identify topics and themes.

**Results:**

According to stakeholders, adolescents have sufficient understanding of the importance of PA and a healthy diet, but nevertheless engage in unhealthy behaviour. Parents were mentioned as important facilitators for engaging adolescents in healthy lifestyle behaviours. Stakeholders listed lack of knowledge, time, and financial resources as barriers for adolescents from low SES families to engage in healthy lifestyle behaviours. The school environment was listed as an important facilitator of adolescents’ healthy lifestyle changes, but stakeholders acknowledged that current school days, curriculum and buildings are not designed to promote healthy lifestyle behaviours. External support and collaboration with community and governmental stakeholders was seen as potentially beneficial to improve healthy lifestyle behaviours.

**Conclusions:**

This study shows the variety of barriers adolescents from low SES areas face, and the need for a broader collaboration between key stakeholders to facilitate healthy lifestyle behaviours. Schools are regarded specifically as important facilitators. Currently, the school environment entails various barriers. However, when addressing those, schools can increase opportunities for healthy lifestyle behaviours of adolescents from low SES areas.

**Trial registration:**

This study is registered in ClinicalTrials.gov on 12/08/2021: NCT05002049.

## Background

Obesity in adolescence has increased over the last decades [1, 2]. In Europe, self-reported data showed that the proportion of overweight or obese adolescents significantly increased between 2018 and 2022. Although differences per country were observed, no countries showed a significant decrease [2]. Furthermore, the majority of adolescents fail to meet the recommended guidelines for physical activity (PA) [3] and a healthy diet [4]. Only 19% of adolescents in Europe engage in an average of 60 min of moderate-to-vigorous PA daily per week [1]. Whilst the World Health Organisation (WHO) recommends to eat 400 g, equivalent to 5 servings, of fruit and vegetables each day [4], still 48% of adolescents do not eat fruit and vegetables on a daily basis [1, 3]. Research shows that adolescents living in areas with a low socioeconomic status (SES) are less likely to have healthy eating behaviours, participate less in PA, and are more likely to be overweight [1].

Several studies have been performed to increase healthy lifestyle behaviours among adolescents, but with limited and inconsistent evidence on effects [5]. Especially adolescents with low SES are understudied in interventions targeting healthy lifestyle behaviours. In line with interventions targeting adolescents in general, interventions targeting adolescents with low SES showed inconclusive results on effectiveness [5–7]. Mixed results have been found regarding dietary behaviours among adolescents from low SES backgrounds [5], but the majority of studies showed at least some improvement [7]. The umbrella review of Craike et al. regarding interventions to improve PA included only three reviews in which just a few studies were effective among adolescents from socioeconomically disadvantaged communities [6]. Previous studies highlight the need for more research on healthy lifestyle behaviour interventions among this target population, with the involvement of adolescents in developing and delivering intervention components being a promising method [6, 7]. This is in line with a statement of the WHO, as they report that one way of creating more effective interventions is to engage young people in developing and implementing sustainable changes, among others in health [8–10]. Citizen science is one approach to collaborate with the target group in various aspects of the research project, which can include defining the problem, developing the intervention, data collection, analysis, and interpretation, and dissemination [11–13].

The Science Engagement to Empower aDolescentS (SEEDS) project aimed to improve healthy lifestyle behaviours in adolescents by using an extreme citizen science approach [14] in developing, implementing, and evaluating healthy lifestyle interventions in high schools in four European countries [15]. Extreme citizen science is a form of collaborative science with a high level of engagement of participants, involving them in various parts of the research which should be focused on their needs [14]. The SEEDS project specifically focused on adolescents growing up in low SES areas. The first step in the approach is to have a better understanding of the needs and experiences from the target group [16]. In the SEEDS project, focus groups were performed to investigate the main barriers and facilitators regarding healthy lifestyle behaviours, mainly focusing on PA and healthy diet, according to adolescents from low SES areas, and among key stakeholders working with this target group [10, 15]. Key stakeholders in the SEEDS project are relevant individuals with various expertise and can play a role in promotion of healthy lifestyle behaviours among youth [17]. They have insights into the underlying factors of healthy lifestyle behaviours among youth and can reflect in a unique way on the intention of adolescents to engage in healthy lifestyle behaviours. Someone’s intention to engage in a certain behaviour is crucial for behaviour change based on the Theory of Planned Behaviour (TPB). The TPB describes that intention is derived from underlying attitudes, subjective norms, and perceived behavioural control [18–20]. Key stakeholders can play an important role in removing barriers within those underlying factors or in facilitating healthy lifestyle behaviours [16, 21, 22].

Previous studies which used existing literature and consultation of adolescents, parents, health professionals and community stakeholders showed the presence of several barriers for adolescents to engage in healthy lifestyle behaviours: personal factors, like motivation, and both social and physical environmental factors, like limited access to sports opportunities or limited availability of healthy food at home [23–25]. Additionally, they mentioned influencing factors from interpersonal to societal levels, including parental involvement, teacher engagement and societal or governmental decisions [23, 26, 27]. Previous research also investigated strategies within different settings to increase healthy lifestyle behaviours, for example, by providing youth sports programmes, ensuring sufficient time to be active during school hours [23], or by promoting healthy lifestyle behaviours through multi component interventions, such as focusing on both schools and family [28].

So far, there is limited evidence on effective interventions targeting healthy lifestyle behaviours among adolescents with a low SES. As this group is less likely to engage in healthy lifestyle behaviours, it is important to identify and understand their barriers and facilitators. The barriers and facilitators regarding healthy lifestyle behaviours among adolescents from low SES areas and the role of stakeholders have not yet been studied in relation to the TPB. Therefore, the aim of this study is to gain insights from key stakeholders on the barriers and facilitators for adolescents living in low SES areas regarding healthy lifestyle behaviours, and to examine the role of stakeholders in overcoming these barriers.

## Methods

### The SEEDS project

The aim of the SEEDS project was to improve healthy lifestyle behaviours in adolescents (13–15 years old) living in low SES areas and to increase their interest in Science, Technology, Engineering and Mathematics (STEM) [15]. A group of adolescents from intervention schools in each pilot country, called ambassadors, were involved in shaping the research questions, designing the interventions and carrying out the interventions in the framework of an extreme citizen science approach [14]. This ensured the adolescents could collaborate meaningfully and share their input to develop and implement healthy lifestyle interventions. The ambassadors were a smaller group of the adolescents using the intervention.

The SEEDS project had pilot sites in Spain, Greece, the Netherlands, and the United Kingdom (UK) and focused on high schools in low SES areas. The indicators used to define these neighbourhoods per country are presented in the study protocol [15]. The trial that was part of the SEEDS project is registered at ClinicalTrials.gov (NCT05002049) on 12/08/2021 and the four pilot countries obtained approval for the study from their corresponding Ethical Committee.

The SEEDS project involved key stakeholders related to the healthy lifestyle behaviours of adolescents in low SES areas, e.g. community partners, school staff and governmental parties. In the initial stages of the SEEDS project, focus groups were planned with adolescent ambassadors to reflect on their healthy lifestyle behaviours and corresponding barriers and facilitators. Although promoting more PA and healthy eating were the main healthy lifestyle behaviours the SEEDS project focused on, adolescents could specify their choice to address an additional healthy lifestyle behaviour in the intervention of their country. Afterwards, key stakeholders reflected in separate focus groups on the factors mentioned by the adolescents and identified their roles in facilitating healthy lifestyle behaviours in adolescence. The results of all focus groups were used to guide the co-creation process, the development and implementation of high school-based lifestyle interventions and specified the main outcomes measured in the SEEDS project.

Following the focus groups, one co-creation event was organized in each country, in which adolescent ambassadors who were supported by key stakeholders in the field of healthy lifestyles, developed intervention activities for increasing PA, improving healthy eating and to address an optional third healthy lifestyle behaviour. Outcomes of the co-creation events defined the country specific interventions, e.g. specific themes, number and type of activities, and the role of ambassadors.

The overall SEEDS project has been described elaborately in a study protocol [15]. This study only reports stakeholders’ views on the two main healthy lifestyle behaviours of the SEEDS project: PA and healthy diet.

### Focus groups

Six semi-structured focus groups were conducted with 28 stakeholders from different settings, such as local schools, the community or the (local) government. At least one focus group per country was conducted and as those focus groups were exploratory for the next phases of the SEEDS project, saturation was not sought. Rather, a good representation of local stakeholders was sought. Depending on research constraints, the number of participants in each focus group differed. However, even a small number of focus groups and participants can yield rich qualitative data and offer deep understanding of participants’ perspectives [29]. In each country, stakeholders were invited to the focus groups based on their expertise of, experience with or influence on healthy lifestyle behaviours of adolescents from low SES areas. They were identified by the research team and by the adolescents who participated in their focus groups. The final selection of stakeholders was representative for the healthy lifestyle behaviours adolescents want to address in the intervention. Participants were recruited either via the research team, the intervention schools or local partners. They did not receive an incentive for participating. Informed consent was obtained for each participant. The duration of the focus groups ranged from 40 to 90 min. Focus groups were audio-recorded, transcribed, anonymized, and translated into English. Focus groups were held between July 2021 and September 2021 and were delivered by a researcher from the respective research institute in person or online, depending on the COVID-19 situation in each country. The theoretical framework of focus groups was developed by the SEEDS consortium and was based on the TPB. This model is structured around three fundamental pillars: (1) the attitude of the person towards the behaviour performed (linked to their individual beliefs around the perceived outcome by the behaviour), (2) the subjective norm (linked to the social environment of the subject) and (3) the perceived control over the behaviour conducted (linked to each individual’s weighted power of intentional behaviour performance) [18–20]. The main questions of the focus groups (Table 1) were based on the TPB and repeated multiple times to separately focus on PA during school hours and healthy snacking choices in and outside school. The SEEDS project focused on behaviour change during one academic year, and therefore focused on a 6 month time window.

**Table 1:** Main questions of focus groups with stakeholders, repeated for physical activity and healthy diet

Pillar of TPB	Question
*Behaviour and attitudes*	• Can you identify any benefits/hazards related to […]?
*Subjective norm*	• What role do friends/family/school/stakeholders play in […]?
*Perceived behavioural control*	• What are the main facilitators and barriers of […]?
	• If you want to change […] as a stakeholder, are you able to? How?
	• Do you think it is reasonable to change (some factors) in […] during a 6-month intervention? How?

Abbreviations: TPB = Theory of Planned Behaviour

### Data analysis

Qualitative data collected in the focus groups were analysed using thematic analysis [30] to identify topics and themes related to PA and healthy diet. All English transcripts were uploaded to NVivo (*version 12*) and reviewed, coded and discussed by two researchers (AW and CME). The steps outlined by Braun and Clark were followed: [1] familiarising with the data, [2] generating initial codes, [3] searching for themes, [4] reviewing themes, [5] defining and naming themes, and [6] producing the report [30]. Steps 1 to 3 were first completed independently to avoid researchers biasing each other, and then discussed together until agreement was reached. The two researchers independently generated codes by open coding using an inductive approach [30]. Subsequently, they compared the codes, looked for similarities, discussed differences, and generated new codes if needed. After the first batch of codes was developed, they independently combined them into major themes and reviewed the developed themes together. An inductive approach for data coding had been used, still the overarching themes were representative of the TPB. Results were narratively described and quotes were added to support findings.

## Results

In total 28 stakeholders participated in the focus groups, of which 13 male and 15 female. The number of focus groups and involved stakeholders differed per country and setting, as explained in Table 2.

**Table 2:** Type and number of stakeholders involved in the focus groups (FG) in each country

Country	Type of stakeholder	Sex	Setting
*Greece* (1 FG, *n* = 5)	• High school principal • Canteen owner of high school • PE teacher • Home economics teacher • Teacher responsible for breaks	• Male • Female • Male • Female • Female	• School • School • School • School • School
*The Netherlands* (2 FG, *n* = 11)	• Municipal project leader for childhood fitness and obesity prevention program • Municipal policy and district advisor on youth • Youth worker at youth foundation • Youth worker at youth foundation • Preventive youth health nurse • Municipal strategic advisor sports • Municipal policy and district advisor on youth • Employee of sport facilitating agency • PE teacher and municipal project leader at school for childhood fitness and obesity prevention program • Youth worker at a welfare organization • Researcher at sport supporting agency	• Male • Male • Male • Male • Female • Female • Male • Female • Male • Female • Female	• Government • Government • Community • Community • Community • Government • Government • Community • School • Community • Community
*Spain* (2 FG, *n* = 10)	• PE teacher in high school and sports campus manager • Policymaker of the health promotion service in a public health agency • Social integrator of a high school • Youth worker • Member of confederation of charitable and social action entities, and retired university professor • Member of the central market • Policymaker of educational services • Pedagogue and expert in health promotion • Council of youth and citizen participation and of rural environment • Teacher at a unit of shared schooling	• Male • Female • Female • Male • Male • Male • Female • Female • Female • Male	• School • Government • School • Community • Community • Community • Government • Community • Government • School
*United Kingdom* (1 FG, *n* = 2)	• Head of science department of high school • Representative of a STEM charity organisation	• Female • Female	• School • Community

Abbreviations: FG = focus group; PE = physical education; STEM = Science, Technology, Engineering, and Mathematics

Three main themes could be identified in line with the TPB. The first theme ‘behaviour and attitudes’ is focused on the benefits and hazards of engaging in healthy lifestyle behaviours and the opportunities stakeholders see to change. The second theme ‘subjective norm’ describes the role and influence of others on adolescent engagement in healthy lifestyle behaviours. The third theme ‘perceived behavioural control’ shows the facilitators and barriers for adolescents engaging in healthy lifestyle behaviours identified by stakeholders.

### Behaviour and attitudes – benefits and hazards of engaging in healthy lifestyle behaviours and opportunities to change

Stakeholders mainly focused on adolescents’ knowledge and less on adolescents’ attitude towards healthy lifestyle behaviours. According to stakeholders, adolescents know the basic benefits and importance of being physically active and having a healthy diet. However, they also indicate knowledge is not enough and highlight that an additional step in creating raised awareness is needed. For example, they are taught about how much sugar is in certain drinks, but is that enough?

*You have to go one step further. 5 sugar cubes*,* what does that do to you? […] What are the dangers when you drink a drink with 5 sugar cubes. Now it is only shown how much sugar is in it. Most young people just think*,* now what? I just like that. Perhaps that is also a point of concern.* (Netherlands, community, male)

Moreover, stakeholders mentioned the broader views they have on benefits of healthy lifestyle behaviours compared to adolescent views, for example on emotional well-being and the long-term effects of healthy lifestyle behaviours. Furthermore, stakeholders see the sustainable engagement of adolescents in these healthy lifestyle behaviours as a major challenge.

*Of course*,* healthy eating habits are established*,* physical education hours are done but the problem is that this does not have an external consistency […] that here they make it “forced” but then they don’t establish it in their day-to-day life.* (Spain, school, female)

Teachers mentioned that many adolescents in their schools skip breakfast, eat unhealthy snacks or consume energy drinks, and although teachers highlight the negative influence of these behaviours, adolescents do not appear to change their habits.

*What I did notice*,* […] those energy cans are really cheap. […] That lowers the threshold for both young girls and young boys to buy such a can. Then you really see children from 10 to 11 years old already walking with such a can or 2 or 3. Sometimes I think*,* what are we supposed to do with this?* (Netherlands, community, male)

Also, there are significant others who might affect adolescents’ habits.

*I can assure you that most pupils do not eat breakfast […] There are the parents who do not have time to fix anything for breakfast and it’s sometimes easier to give money to their children […] There is the grandma at home who is in charge of the child’s diet […] they overfeed the children to have them be full of energy and make sure the parents are calm that their kid can eat well. […] What can we do to fix this? It’s something that has multiple holes*,* if you know what I mean.* (Greece, school, male)

Therefore, when targeting healthy lifestyle behaviours in interventions, stakeholders think it is important to be realistic about the results you can achieve in a certain amount of time. Stakeholders from various fields mentioned that first steps can be taken in just a few months.

*To introduce and give information to see […] some changes*,* of course. Anything that breaks the usual routine and could make it easier […] but big changes need more time.* (Spain, government, female)

All stakeholders agreed that behaviour change takes time and more time is needed to create changes in habits or certain behaviours. They also shared the importance of having to start somewhere when you want to make a change, because it is important to keep promoting healthy lifestyle behaviours among adolescents.

### Subjective norms – the role and influence of others on engaging in healthy lifestyle behaviours

Parents, peers, role models and teachers have been identified by stakeholders in all focus groups as people playing a big role and influencing adolescents’ healthy lifestyle behaviours. SES of the family is seen as a barrier.

#### Parents & SES

Creating awareness about healthy lifestyle behaviours might be a first step to change, but adolescents are only one part of this. Stakeholders noted that the lack of time and knowledge of parents is an issue.

*The parents […] have an enormous influence on those children*,* of course. If those children will get food from home that is not healthy*,* then things will go wrong.* (Netherlands, community, female)

Therefore, educating parents about what is healthy can be an important aspect in improving adolescents’ healthy lifestyle behaviours, because adolescents often bring food from home bought by their parents. Parents are seen as important facilitators for having a healthy diet and can also encourage their children to be physically active. However, one major barrier identified by stakeholders is the socioeconomic environment of the family.

*Healthy food is still more expensive than unhealthy food. So also some parents*,* who choose the easier way. At least*,* the way that costs the least money.* (Netherlands, community, female)

*Because many times who has access to the healthiest things is who has more socioeconomic power*,* who has more level*,* who can go to the gym or who can buy a quality vegetable […] And on the other hand*,* […] in more disadvantaged areas it is much more difficult to access all these options.* (Spain, government, female)

Stakeholders mentioned that parents might even have the knowledge or willingness to empower their children to engage in healthy lifestyle behaviours, but that their SES and mainly income-related issues, like expensive food or sports memberships, remains an important barrier to being able to translate knowledge into action.

#### Peers & role models

Stakeholders mentioned the major influence friends have on one another, both positively and negatively. *‘When one stops*,* then the other stops’* (Netherlands, community, female). This is particularly the case for PA. Peers can act as both barriers and facilitators to participation in physical education (PE) class, activities during breaks or extracurricular sports. Stakeholders think leaders are needed to encourage healthy lifestyle behaviours.

*In the school situation. Perhaps also peers*,* so someone from a higher year or someone who is good at what he does. Maybe not in sports per se*,* but in connecting. That does help.* (Netherlands, school, male)

This leader can be another student, but according to stakeholders there are also others who influence adolescents’ lifestyles. One example is influencers promoting various foods and drinks on social media, even though this may not always be the healthiest option. Famous athletes or sport coaches can also encourage different people, as preferences differ within various age groups and between boys and girls.

*That is also positive*,* if you also have a mix in sports coaches. Because they also want to identify with someone. With whom they also feel safe.* (Netherlands, community, male)

Whether this role model is a peer, a sports coach in the neighbourhood or a social media influencer, stakeholders think they can all encourage more participation in healthy lifestyle behaviours.

*There should be someone they believe in and can identify with. That’s a really important starting point*,* if you want to get inside someone’s head and really change something.* (Netherlands, government, male)

#### Teachers

As adolescents spend a lot of their time at school, teachers can also be seen as role models. Teachers themselves mention they can encourage students in healthy lifestyle behaviours by setting a good example or applying rules at school. However, there is also a limit to this. *‘We don’t want to fall into policing it’* (United Kingdom, community). At the same time, teachers are expected to model healthy lifestyle behaviours. Whereas some teachers are open to trying new ideas, others are a bit hesitant to let students be more active at school.

*A teacher […] has also completely redesigned a room to tempt them [the students] to move more during the lesson. But you notice that teachers are not ready for that yet. Because a lesson becomes more restless*,* of course*,* if more movement is allowed there. And how are you going to organize your lesson accordingly?* (Netherlands, government, male)

Teachers try to keep classes fun and interactive for their students with regards to nutrition and PE classes. They think PE class is a way of relaxing, playing and having fun with classmates, as well as having social contact and ensuring inclusion of all groups, which is also acknowledge by other stakeholders.

*I believe that PE lesson is important for the pupils to relax from the routine of school*,* have fun and play. Of course*,* I believe they can also learn to work as a team*,* gain discipline*,* become stronger in body and mind. These things need to be taught and sculpted as well you know. These are even more important than how to teach a kid to do a volleyball shot properly.* (Greece, school, male)

*Through [physical] activities*,* especially when we talk about vulnerable groups*,* who are probably the ones who have the most problems with sedentary lifestyles because they are immersed in less activities*,* inclusion is generated as well as a space for group relationships*,* which is so important in adolescence and the early stages of youth.* (Spain, government, female)

Teachers do give personal opportunities for their students and encourage them to participate during PE class. However, it is more difficult to affect their PA outside PE lessons and their dietary behaviours.

### Perceived behavioural control – facilitators and barriers for engaging in healthy lifestyle behaviours

Various barriers and facilitators regarding school, support and collaboration, which make it difficult to engage in healthy lifestyle behaviours for adolescents, have been discussed by stakeholders.

#### School

While teachers mention their facilitating role in engaging students in healthy lifestyle behaviours, the school environment provides various barriers that do not enable this. Overall, the structure of the school day and the curriculum mainly results in sedentary time and reducing this is a challenge. Furthermore, the curriculum is full of compulsory parts. Teachers say they are willing to focus more on healthy lifestyle behaviours during their lessons and they expect that nutritional education might positively affect the food choices of adolescents. However, demands are high and the schedule does not always allow for this.

*I have tried to tell them about nutrition. It is totally linked with my lesson. But when? […] There were these “projects” that were organized for some years […] involving nutritional issues but were inconsistent and we didn’t always have the right person to organize them properly. The biggest issue is always time.* (Greece, school, male)

However, not only during the lessons, but also during breaks or after school time, there is a lack of opportunities to engage in healthy lifestyle behaviours at high schools according to stakeholders.

*You go out from primary school running. Then you come to secondary education*,* there you have a square and then you start chatting. Then you sit*,* then you hang. Then you are in a group. But it’s not really normal there to play basketball or something like that.* (Netherlands, community, female)

Breaks are short and most adolescents will socialize and eat with friends instead of being active. Longer breaks could be a solution to be more active, but stakeholders mention the need for facilitators, people supporting healthy lifestyle behaviours, to empower adolescents to do so and facilities or space to play sports being available.

*Activities at lunch aren’t focused – kids occupy themselves - there isn’t enough space. Kids would engage with things if it were structured and organized. If it is football they will do it – they can organize it themselves. Engagement in other clubs is a small minority – school is the biggest opportunity for them.* (United Kingdom, school, female)

At the moment, schools are not designed for being active, while it can actually be an opportunity to engage in healthy lifestyle behaviours. If adolescents engage with various sports during school hours, the threshold for participating in PA outside of the school environment can be lowered.

*We have [partners] in our neighbourhood […] who get to work with the children more actively during the break. […] [We are also] bringing the schools to the houses of the neighbourhood. To actually introduce the young people to the low-threshold activities in houses of the neighbourhood*,* so that it is actually easier for them to go to the gyms […] and with the possibility of outflow to the sports organizations in the area. […] The sports that they don’t normally come into contact with.* (Netherlands, community, female)

However, stakeholders are also aware of the various barriers faced for PA outside of school.

*I think we are wrong when we say that it is an economic issue only and we mark it with that because it is often more cultural or access issue or support beyond the economic.* (Spain, community, male)

Regarding healthy eating at school, the canteen and the policy on healthy foods at schools are seen as important factors. For example, some schools have a ban on energy drinks and others forbid students to leave school during breaks.

*I think the school’s policy is important. From what age are they allowed to leave the schoolyard at all during recess? […] The difficult thing is often what is available near the school.* (Netherlands, government, female)

When students are not allowed to leave school during the school day, they eat and drink what they bring from home or they rely on what is available at the school canteen. However, then the offer should be made healthy as well.

*What you do notice at the schools is that the products offered are very limited. […] To be fair*,* we get fruit for activities. But if you will get something at the [canteen] counter*,* it’s candy because that will sell. But I do notice in more and more programs that we have done in which healthy food is used very consciously*,* that at a certain point the children also start asking for it. So I think it really has to do with the products offered.* (Netherlands, community, female)

Aside from school canteens still offering a lot of unhealthy food, price of the food plays an important role as well. Overall, stakeholders mention that unhealthy food is less expensive and that especially in low socioeconomic areas this issue is important in diet choices.

*Kids prefer the tastier snacks and those that keep them full. A cereal bar costs more than a cheese pie and may be less tasty and not suffice for a whole breakfast. I tell you*,* I am doing this job for many years*,* I have seen what is sold and what is not.* (Greece, school, female)

Although some canteen owners are able and eager to try introducing healthy products in school canteens, they recognise changes do not last, as students are not buying those healthier products for the long-term.

*I sure did try it. There was a time when I thought that fruit salads would be great for children. I ordered various fruits […] and put them in plastic packs for the kids to try. During the first 2 weeks the kids were fascinated by the new product. They were always sold out. After these two weeks*,* the fruit salads sales dropped dramatically and finally they did not sell at all.* (Greece, school, female)

#### Support and collaboration

Overall, stakeholders mentioned that school can be an important facilitator for adolescents to engage in healthy lifestyle behaviours, but in reality, this is not always the case. Support from other teachers or the school management is mentioned to be empowering for teachers, but stakeholders also say that more support and collaboration is needed from outside the school, in the community surrounding adolescents.

*The high school should not be 100% responsible for this being generated*,* but the high school is part of a community*,* is part of a group that must be able to generate these dynamics. […] The high school […] cannot be able to generate these activities autonomously. Therefore*,* if they do not have the resources to generate these types of activities*,* the student will not do it.* (Spain, government, female)

Stakeholders also mentioned that students are more motivated to participate in healthy lifestyle behaviours when they are rewarded for their positive behaviour, for example incentivizing adolescents with a big sports event at school when they participate well in PE class. However, teachers do not always see the direct effects of those rewards.

*It is good to link up with […] what do you want to achieve? But of course also with the young people*,* because they don’t run faster because of all the rewards that we come up with. But it really does have an effect. In the long term*,* however*,* you can give shape to a behavioral change.* (Netherlands, school, male)

In the end giving rewards for healthy lifestyle behaviours of adolescents is seen by stakeholders as being part of creating a change in their behaviour, becoming part of adolescents’ intentions to live healthier and facilitating healthy habits.

To be able to make a difference, the environment around the school is another important factor discussed by stakeholders when influencing the engagement of adolescents in healthy lifestyle behaviours. Shops around school will offer cheap and unhealthy snacks and facilities or spaces in the neighbourhood might not be suitable or available for adolescents’ physical activities.

*So I think it’s not that there aren’t sometimes people who want to play sports*,* but maybe sometimes there aren’t all the facilities or all the places to address those needs.* (Spain, community, male)

Adolescents would be willing to participate in after school sports clubs and with some support from, for example, neighbourhood facilities, youth workers or the municipality, those opportunities can be realized according to stakeholders.*‘Generating healthy spaces*,* […] it’s a whole-of-society issue.’* (Spain, community, male).

The specific role of stakeholders in this study with regard to facilitating healthy lifestyle behaviours for adolescents varied per setting. Changing the products in school canteens, setting rules at school, modelling healthy lifestyle behaviours, or providing sport workshops are direct ways to facilitate healthy lifestyle behaviours, primarily by stakeholders in the school setting. Community and governmental partners were more likely to make indirect changes, for example, by designing policies or school health programs. Therefore, all stakeholders see collaboration within the community as key to creating a healthy environment that empowers engagement in healthy lifestyle behaviours.

## Discussion

This study provides insights from stakeholders across four European countries on the barriers and facilitators for adolescents from low SES areas regarding healthy lifestyle behaviours. This study also focuses on the possible role of stakeholders in overcoming barriers and facilitating healthy lifestyle behaviours. To the best of our knowledge, this is the first study to investigate stakeholder perspectives on healthy lifestyle behaviours of adolescents living in low SES areas, an underrepresented group in research. According to stakeholders, adolescents acknowledge the importance of PA and a healthy diet, but still do not engage in healthy lifestyle behaviours. Parents, peers, role models and teachers influence healthy lifestyle behaviours of adolescents, both positively and negatively, and family SES is seen as an important barrier for engagement in healthy lifestyle behaviours. Mainly the school environment was mentioned as a barrier to both PA and healthy dietary behaviours. External support from and collaboration between different stakeholders was mentioned as facilitating for healthy lifestyle behaviours of adolescents. When barriers will be addressed, school were seen as an important setting to target all adolescents.

In our study the TPB framework was used. Whereas attitude in the TPB framework more broadly focuses on an individuals’ attitude towards a certain behaviour [18], stakeholders in our focus groups specifically focused on knowledge. They acknowledge adolescents have basic knowledge about healthy lifestyle behaviours, but at the same time show unhealthy habits. Adolescents, parents, and community members in the American community-based participatory research of Goh et al. mentioned a relative lack of knowledge of healthy dietary behaviours of both adolescents and their parents [31]. Although the knowledge levels of adolescents in both samples is different, we agree with the authors it is important to raise awareness and increase motivation of adolescents to change their behaviour and empower healthy habits [31] as increasing knowledge is not sufficient.

When discussing subjective norms, stakeholders highlighted the important role of teachers in facilitating healthy lifestyle behaviours. Whereas peers, role models and especially parents were acknowledged as having both positive and negative influences on healthy lifestyle behaviours of adolescents with low SES. The important role of parents, especially in eating behaviour, was also a common finding in other studies among parents and adolescents [24, 31]. The systematic review by Alliott et al., divides qualitative data on PA barriers and facilitators by SES. They showed that specifically adolescents with a low SES need to rely more on support from friends and teachers, as there are other priorities in their family, like household chores or taking care of siblings [32]. Although those family priorities were not specifically mentioned in our study, stakeholders perceived parental lack of time, money and facilities as barriers, whereas teachers were mentioned to have an encouraging role in adolescents’ healthy lifestyle behaviours. Therefore, healthy lifestyle behaviours should be promoted independent from the available resources at home for adolescents with a low SES.

For all adolescents, social media influencers can affect their healthy lifestyle behaviour. Although recent literature mainly focused on the negative impact those influencers have on adolescent’ health, like promoting unhealthy diets or giving inaccurate advice [33], stakeholders in our study acknowledged that influencers can also encourage participation in healthy lifestyle behaviours. The scoping review of Engel et al. showed that research on the potential benefits of social media influencers on health promotion remains limited, although positive impact has been seen in for example public health campaigns or by providing support for health issues [33].

Regarding perceived behavioural control, various barriers and facilitators have been identified by stakeholders. The school environment is seen as a major factor influencing healthy lifestyle behaviours in adolescence. Specifically, stakeholders mentioned limited opportunities to be active within and outside school and unhealthy school food environment as barriers of schools. In all four countries, stakeholders mentioned a lack of time for healthy lifestyle behaviours in the curricula of schools. Previous qualitative research shared the same finding among key stakeholders that school often is a barrier for PA and a healthy diet [31, 34], although our study specifically focused on the perspectives for adolescents from low SES areas. Current school days and school buildings are not designed to promote healthy lifestyle behaviours, instead they more seem to support sedentary lifestyles.

Furthermore, key stakeholders mentioned that focus should not solely be at school level. External support and collaboration with community and governmental partners was also considered essential to facilitate healthy lifestyle behaviours among adolescents with a low SES. The importance of a broader collaboration regarding healthy lifestyle behaviour in adolescents was also mentioned in various qualitative studies on determinants of healthy lifestyle behaviour in Portugal, the UK and America [31, 34, 35]. Health education teachers in Portugal mentioned focus is needed not only at school level, but also on community and political level [35]. Local community centres were seen as facilitators by adolescents with a low SES [32]. In our study, key stakeholders also reflected on their own role in facilitating healthy lifestyle behaviours among adolescents. Primarily stakeholders from the school setting saw their potential role to directly affect healthy lifestyle behaviours, whereas community or governmental partners saw a more indirect role in facilitating healthy lifestyle behaviours.

Low SES of the family was recurrently mentioned by the stakeholders as a barrier for adolescent engagement in healthy lifestyle behaviours. The review by Alliott et al. showed that adolescents with a low SES mentioned, among others, the lack of money and time of parents, and the lack of facilities and safety as reasons to not participate in PA, whereas adolescent with middle and high SES did not mention any of those barriers [32]. Key stakeholders in our study also highlighted some of those barriers, such as lack of money, time and facilities. Moreover, our study focused on dietary behaviours, and lack of money was seen by stakeholders to greatly influence adolescents’ food choices.

### Strengths and limitations

There are a number of strengths and limitations of this study. Strengths include the novel multi-country perspective of key stakeholders from different settings on the same issues regarding healthy lifestyle behaviours of adolescents from low SES areas in high income countries. The main barriers and facilitators do not seem to differ between different countries. The use of the TPB framework enabled the gathering of barriers and facilitators on different levels, ranging from (inter)personal to environmental factors. Limitations are the relatively small number of focus groups conducted, although there was overlap for many topics in the different focus groups. As this was an exploratory study, saturation was not sought. A variety of external stakeholders were involved in this study, despite the fact that in some countries individuals from the community and governmental setting were less represented. This makes it more difficult to generalize these results. Lastly, in this study we did not specifically focus on the sex and gender differences of adolescents’ healthy lifestyle behaviour, despite previous literature showing the importance of tailoring interventions to the different behaviours of boys and girls [31, 36].

### Implications

The findings of the SEEDS focus groups had practical implications in the consecutive co-creation and implementation of the school-based citizen science healthy lifestyle behaviour interventions in each country. The co-creation event was focussed on PA, healthy eating and an optional third behaviour chosen by adolescents. Key stakeholders who were identified in the focus groups supported the adolescents in the development of intervention activities during the co-creation event. Although the adolescents were in the lead of designing activities, all countries focused on topics that were also discussed in the stakeholders’ focus groups. For example, in every intervention there is a focus on the healthiness of the school environment, either during PE class, in the school canteen, during breaks or during classes.

In general, the practical implications of our findings highlight that more bold initiatives to transform schools into health-promoting sites are needed. The perceived basic knowledge of adolescents on healthy lifestyle behaviours seems not enough, therefore other strategies supporting behaviour change are needed. One of those could be to raise awareness on adolescents’ own eating habits and the long-term effects of unhealthy behaviours. Interviews with adolescents could report on their experiences with and attitudes towards healthy lifestyle behaviours and the perceived influence of teachers on their healthy lifestyle behaviours. Furthermore, more opportunities to increase healthy lifestyle behaviours among adolescents with a low SES may be facilitated independent of the resources available at home, such as parental time or money. This can be achieved by a close collaboration of different parties, including parents, schools, and local community members, for example, to engage adolescents in PA outside of school hours. The findings of this study show that the role of teachers and collaboration with stakeholders is important in implementing interventions to support healthy lifestyle behaviours among adolescents. Therefore, future research should focus on the role of teachers and stakeholders in implementation of healthy lifestyle behaviour interventions by mapping effectiveness, success factors, barriers and facilitators.

## Conclusion

The current study showed stakeholder perspectives on barriers and facilitators regarding healthy lifestyle behaviours of adolescents at (inter)personal and environmental levels. According to stakeholders, adolescents acknowledge the importance of PA and a healthy diet, but still do not engage in healthy lifestyle behaviours. Parents were mentioned as important in engaging adolescents in healthy lifestyle behaviours, but low SES remains an important barrier to facilitate healthy lifestyle behaviours. The school environment was listed as an important facilitator of adolescents’ changes in healthy lifestyle behaviours, but stakeholders acknowledged that current school days, curricula and buildings are not designed to promote healthy lifestyle behaviours. Approaches that involve external support and collaboration with community and governmental partners in the school setting are seen as promising to improve healthy lifestyle behaviours, especially in the long run for adolescents from low SES groups. School-based lifestyle interventions with stakeholder engagement can be a starting point for creating a change in healthy lifestyle behaviours of adolescents.

## Data Availability

The dataset generated for the present study will not become publicly available. Stakeholders could indicate whether their data could be used for future research outside the SEEDS project. These data are available from the SEEDS consortium upon reasonable request and with permission of the Management Board. The corresponding author can be contacted for this: w.jansen.1@erasmusmc.nl.
